# Clinical evaluation of a patient participation assessment system for upper extremity rehabilitation exercises

**DOI:** 10.1007/s11517-023-03014-7

**Published:** 2024-01-17

**Authors:** Erkan Ödemiş, Cabbar Veysel Baysal

**Affiliations:** Department of Biomedical Engineering, Çukurova University, 01330 Saricam, Adana Turkey

**Keywords:** Participation assessment, Clinical trial, Rehabilitation exercises, Physiological responses, Frozen shoulder syndrome

## Abstract

In conventional and robotic rehabilitation, the patient’s active participation in exercises is essential for the maximum functional output to be received from therapy. In rehabilitation exercises performed with robotic devices, the difficulty levels of therapy tasks and the device assistance are adjusted based on the patient’s therapy performance to improve active participation. However, the existing therapy performance evaluation methods are based on either some specific device designs or certain therapy tasks, which limits their widespread use. In this paper, the effectiveness of a participation assessment system, which can evaluate patients’ therapy performance, tiredness, and slacking independent of any device design and therapy exercise, was clinically tested on ten patients diagnosed with frozen shoulder syndrome. The patients performed exercises using the system once a week throughout their 4-week treatment period. Multiple clinical measurements and scales were employed during the clinical study to assess patients’ progress and status, such as tiredness throughout the therapy process. The clinical data, along with the patient findings obtained from the participation assessment system, were statistically analyzed and compared. The findings revealed that the patients’ improvements and progress during the therapy process clinically coincide with the variations in the performance evaluation results of the system, and the implemented method successfully assesses the patients’ participation during the rehabilitation exercises.

**Graphical Abstract:**

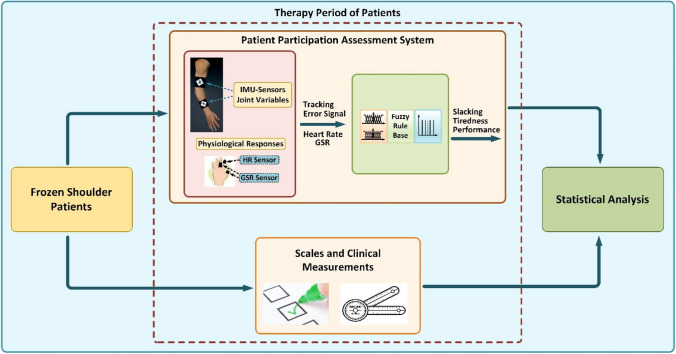

## Introduction

Neuromuscular disorders caused by some reasons such as stroke and spinal cord injury (SCI) severely affect the patient’s ability to perform activities of daily living (ADL) [1, 2]. Conventional therapy, in which exercises are performed with therapists, is one of the most widely used methods for treating patients with neuromuscular disorders. However, this approach has some shortcomings, such as insufficient time being spent with the patient or reduced performance of therapists due to work overload. On the other hand, performing exercises with robotic devices has appeared as a new approach to overcoming the disadvantages of conventional rehabilitation and providing challenging, high-density, and adapted therapy exercises [3–6].

During the treatment process, the patient’s active participation in the exercise is a crucial factor. With the active and voluntary involvement of the patients, maximum functional output and improved neural plasticity could be obtained from therapy [3, 7]. In the rehabilitation exercises with robotic devices, to ensure patients’ active and voluntary participation, the assist-as-needed (AAN) paradigm has emerged [8]. In the AAN approach, the assistance to be applied to the patient and therapy task difficulty level are determined based on patient performance and participation. AAN strategies can be categorized into three main groups [9] as performance-based [10–15], stiffness-based [16–18], and model-based [9, 19–23]. At the heart of the AAN strategies lies the patient performance evaluation method, which serves as the foundation for determining the level of robotic assistance provided to the patient and for adjusting the difficulty level of therapy tasks.

In the literature, many different patient performance and participation evaluation methods have been implemented [10, 15, 20, 23]. However, the existing methods have three main disadvantages: being dependent on specific robotic device design [20, 21], applicability only to certain therapy tasks [13, 15], and neglecting the changing motor capabilities of the patient during the rehabilitation exercises [10, 23]. Additionally, the effectiveness of most methods has not been clinically tested either [24–26].

Papaleo et al. [11], proposed a patient-specific adaptive therapy for an upper limb robotic rehabilitation device, including a module for assessing patient biomechanical performance and a module for adjusting the robotic assistance. The parameters of this approach defined as discrete values and updated when a therapy task is completed. That limits the system’s adaptability and ignores the patient’s changing capabilities. Lin et al. [27], proposed an AAN strategy for wrist exercises that allows passive, active, or resistive rehabilitation. However, the parameters of this method are manually determined and are not tailored to the individual patient. Asl et al. [28], proposed a field-based AAN scheme by building a desired velocity field around the reference trajectory and having a force field to avoid significant position errors. In another study, Zhang et al. [29] presented an AAN strategy that gives patients spatial freedom through a virtual channel around the predetermined exercise trajectory. With these approaches, the constructed velocity field, and virtual channel are not patient-specific and can be limiting for capable patients.

Azlan and Lukman [30] presented an assist-as-need approach for upper limb rehabilitation exercises like basic motions in eating activities. In this study, the patient’s capabilities and impairment level were assessed based on the patient’s exerted forces, by using a force sensing resistor (FSR). Also, some studies have used specific indices based on the performance or experimental measurements of the patient to evaluate their therapy engagement [13, 31]. Mounis et al. [32] proposed a functional ability index based on the wolf motor function test and tested the effectiveness of this method on three hemiplegic patients. In another study, subjects’ motor performance was evaluated using a functional activity spline function (FASF) based on trajectory error, velocity, and time indexes, and the approach was clinically tested on fifteen patients [33]. The primary drawback of these methods is the need for adjustment based on exercises or their inability to adapt to every therapy task. In another approach, an AAN controller scheme focused on therapy task completion, even when the patient is incapable of completing the desired exercises was introduced [34]. In this approach, two different methods were implemented, the force sensor-based method and the disturbance observer-based method. This method also includes an impedance modulation based on patient trajectory tracking performance and interaction forces for improving patient participation. In another approach, [21], a dynamic model of the arm and real-time measurements of the subject’s torque were employed to assess patient interaction forces. Also, this method was clinically tested with six patients. The main disadvantage of these approaches is the performance of the system relies on the measurement quality of patient inputs or precise knowledge of the robot dynamics.

In some studies, patient performance was evaluated only based on the position error signal occurring during the therapy exercises. Zhang et al. [35] presented a control strategy for meeting the therapy requirements of patients in different rehabilitation stages, which includes zero interaction force (ZIF) mode, AAN mode, and restriction interaction region (RIR) mode. In this approach, therapy mode transitions and patient performance evaluation were performed using the trajectory tracking error signal. Lou et al. [9], proposed a greedy AAN scheme that utilizes the radial basis function to model the changing functional capability of patients based on tracking errors due to its simple computational complexity. The authors also performed clinical experiments on 12 subjects with neurological impairments to demonstrate this method’s effectiveness. Also, Li et al. [36] presented a hybrid controller that contains assistive, resistive, and restriction therapy strategies. In this controller, the patient’s capabilities were evaluated based on trajectory-tracking error signals. Depending on the tracking error, the stiffness coefficient of the controller was adjusted using a fuzzy logic. However, evaluating patient capacity or therapy performance only based on the tracking error signal may lead to false assessments in cases where the error signal will reach a small value due to the robotic assistance, despite a lack of the patient's motor skills.

To overcome the disadvantages of the existing studies mentioned above, in our previous work [37], we proposed a new participation assessment system that evaluates the therapy performance of patients and other factors affecting the therapy participation, such as tiredness and slacking, independently from any rehabilitation device design or therapy task. The designed system evaluates the patient’s changing capabilities and therapy participation using physiological responses and trajectory-tracking error signals. This method is cost-effective, portable, and can be used with or without any therapy device. In the system, an upper limb kinematic module has been utilized to model patients’ upper limb movements and physiological responses have been monitored using sensors. A fuzzy inference system (FIS) was implemented to assess to therapy participation of the patients based on physiological responses and trajectory-tracking error signals. Also, the developed method adjusts the therapy tasks and task difficulty level according to the subject’s performance and tiredness. The effectiveness of this system was demonstrated experimentally with healthy subjects on five different therapy tasks [37].

In this paper, the developed participation assessment system was tested on patients with frozen shoulder syndrome (FSS) in clinical trials. Since the designed system is hardware-independent, during the clinical trials, it was implemented without using any therapy devices. In the clinical experiments, patients performed rehabilitation exercises using the system once a week throughout their 4-week therapy period. For demonstrating the developed system’s effectiveness, the clinical study was structured based on comparing the participation and performance assessment data of the designed system with the patient’s progress during the treatment process. In this context, several scales and clinical measurements were performed to evaluate patients’ improvements during the therapy process and their status (like tiredness and slacking) during the use of the developed system. The scales and clinical measurements, in conjunction with the findings procured from the system, were statistically analyzed and compared to provide a comprehensive evaluation. In summary, the main contributions of this study are clarified below:

- The effectiveness of a system that evaluates patients’ active participation in therapy, which significantly contributes to enhancing the functional outcomes of rehabilitation exercises, is demonstrated through clinical trials.
- The device design and therapy task-independent structure of the proposed system is introduced by implementing the method to three different therapy tasks without the use of any therapy device during the clinical experiments.
- The findings obtained from clinical trials reveal the efficacy of using physiological responses and trajectory-tracking error signals to evaluate patients’ participation and performance in therapy exercises.

The remainder of this paper is organized as follows: Sect. 2 explains the overview of the implemented patient participation estimation system and the details of the clinical trials. The results of the experiments with patients and statistical findings are given in Sect. 3. Subsequently, the discussion is provided in Sect. 4. In Sect. 5, the limitations of the study are indicated. Finally, the conclusion is presented in Sect. 6.

## Material and methods

### Overview of the implemented participation assessment system

The implemented system evaluates the patient’s changing capabilities and therapy performance during upper limb rehabilitation exercises based on a multimodal sensor fusion formed by the trajectory tracking error signal and the patient’s physiological data (heart rate and skin conductance) [37]. The developed system also assesses the patient’s tiredness and slacking, which are substantial factors affecting the therapy performance. The novelty of the method is evaluating patient performance independently from any therapy tasks or rehabilitation device designs. Therefore, the system can be applied to any kind of therapy task and is suitable for performance assessment in conventional therapy and sports exercises. Also, the applied method can be used in both clinical and home settings, and it offers a portable and customer-mate solution to assess user participation during the exercises.

**Fig. 1:**
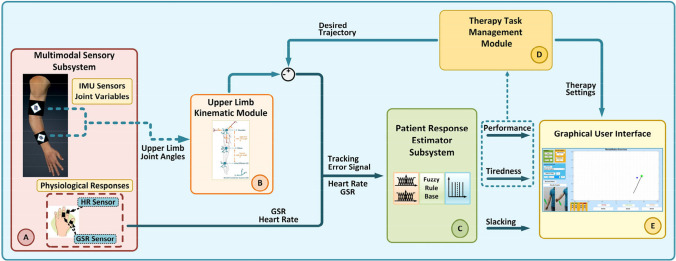
Overview of the implemented participation assessment system. (A) Multimodal sensory subsystem, (B) Upper limb kinematic module, (C) Patient Response Estimator Subsystem (PRES), (D) Therapy task management module, and (E) Graphical User Interface (GUI)

The implemented participation assessment method in this study is a comprehensive system consisting of five subsystems, as illustrated in Fig. 1.

The multimodal sensory subsystem incorporates two types of sensory data; the patient’s upper limb joint angles and physiological responses, such as heart rate (HR) and skin conductance (or galvanic skin response (GSR)) signals. During rehabilitation exercises, two inertial measurement unit (IMU) sensors, one for the upper arm and one for the forearm, are placed on the patient’s upper limb using cuffs to measure the joint angles. Physiological signals are measured using the HR and GSR sensors. These sensors are placed on the patient’s left hand, as seen in Fig. 1. All the sensor data is transferred to the target PC via serial communication with a 10-Hz sampling rate using an Atmel microcontroller (MC) board as a data acquisition system and processed in a MATLAB®/Simulink model.

**Fig. 2:**
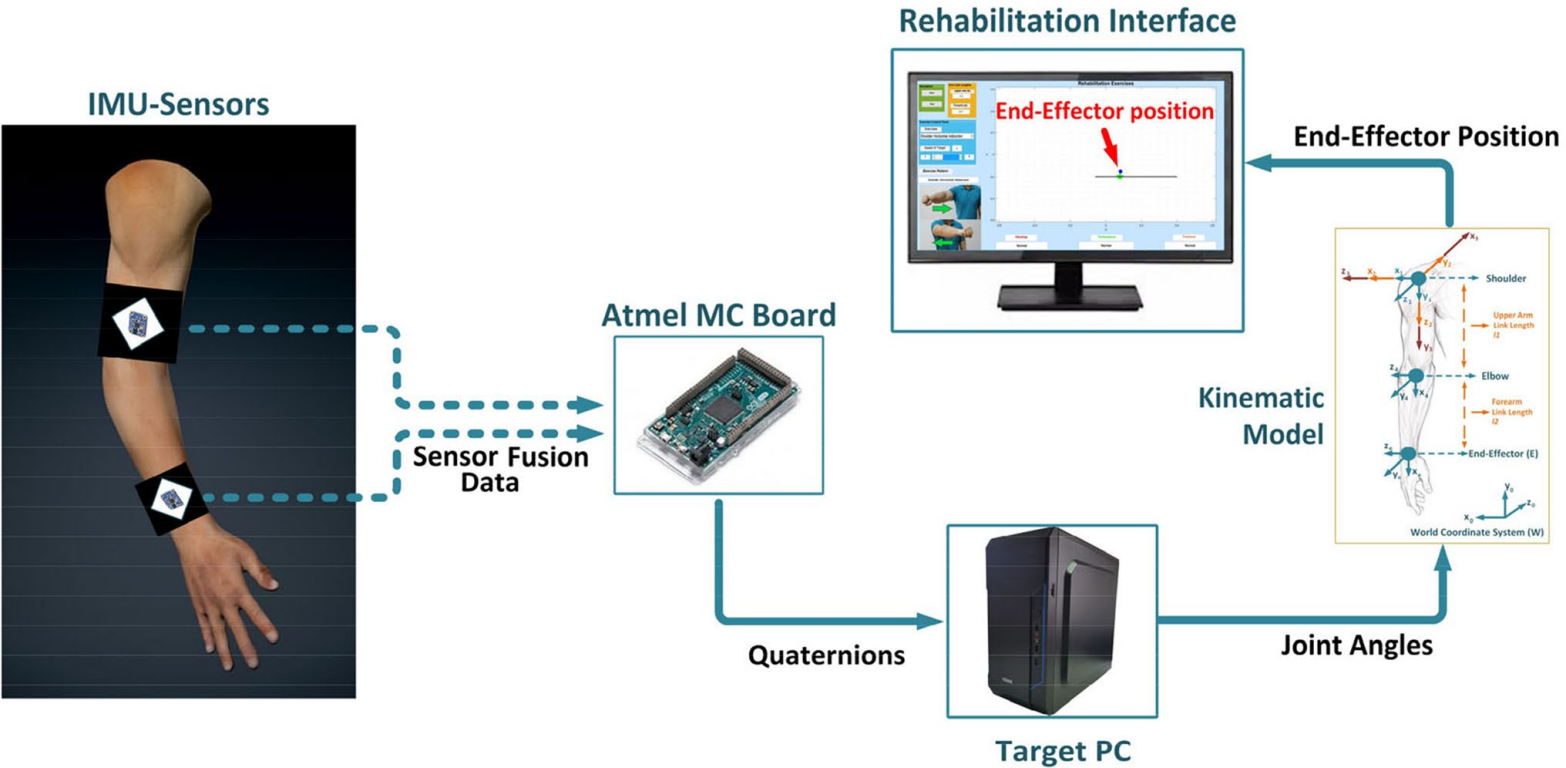
Modelling the patient arm movement during the exercises

The patient’s upper limb movement during the rehabilitation exercises is modeled by using the 4 degrees of freedom (DOF) human arm kinematic model that has 3 DOF at the shoulder (flexion/extension, abduction/adduction, and internal/external rotation) and one DOF at the elbow (flexion/extension). In the implemented arm model, the end-effector represents the wrist of the subject. During the estimation process of the patient’s upper limb motion, firstly, the sensor fusion data of the IMU sensors is transferred to the target PC as quaternions which are four-element vectors used to describe any rotation in a three-dimensional coordinate system [38]. Then, the quaternion values are converted to joint angles [39]. In the final step, the patient’s arm movement is modeled using the human arm kinematic model for performing the rehabilitation exercises and determining the trajectory tracking error signal during the experiments, as seen in Fig. 2. The trajectory tracking error signal expresses the difference between the trajectory target of the therapy task and the patient’s end-effector position and is calculated by comparing these two data during experiments.

**Fig. 3:**
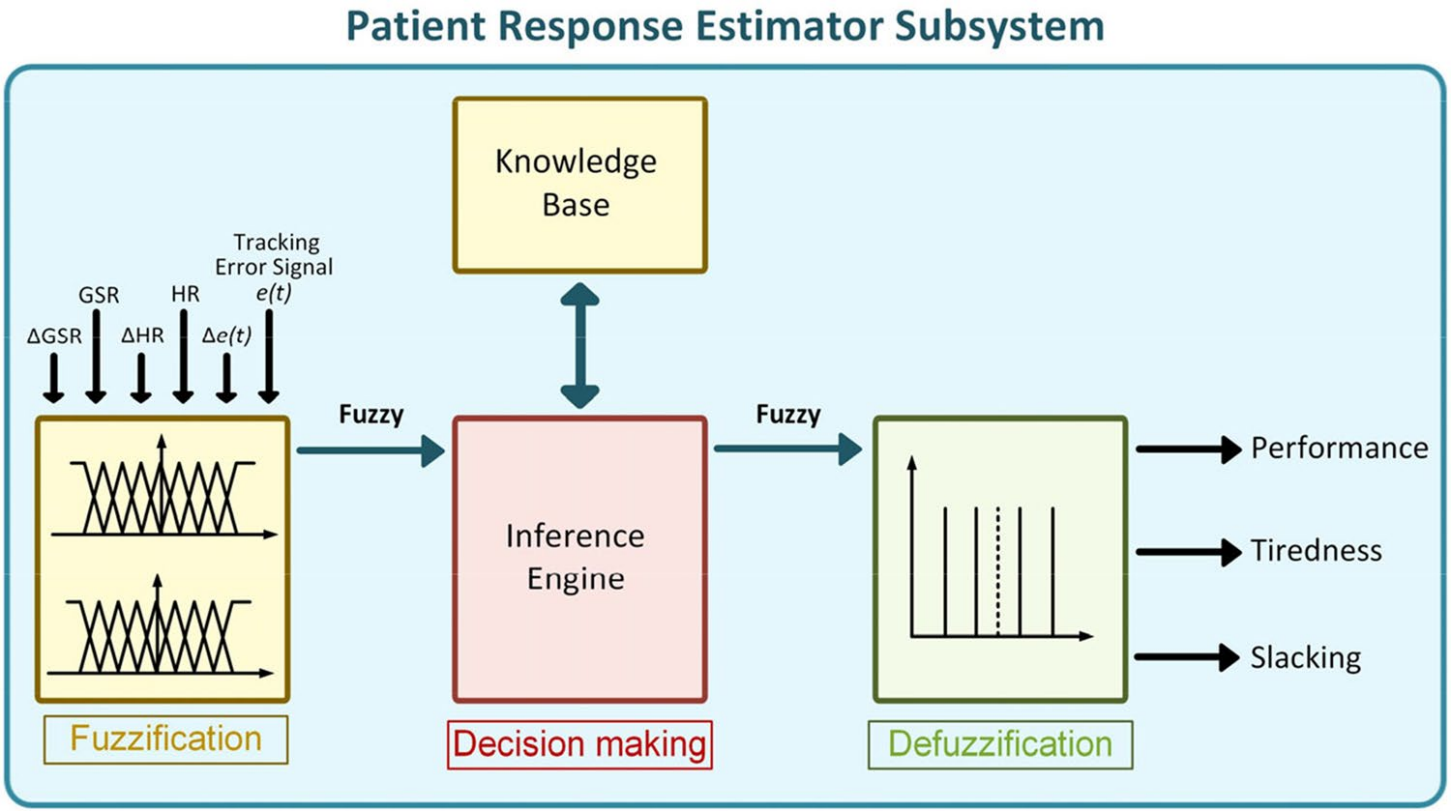
Patient response estimator subsystem

In the patient response estimator subsystem (PRES), a FIS has been implemented for evaluating the therapy performance, tiredness, and slacking of the patients during the rehabilitation exercises. The designed FIS has six inputs: HR, skin conductance, the desired trajectory tracking error, and variation of these signals during the therapy exercises. Physiological responses (such as heart rate, skin conductance, respiration, and skin temperature) are reliable indicators for assessing a patient’s physical activity and emotional state during rehabilitation exercises [40]. Thence, HR and GSR signals are used in the implemented system for evaluating the patient’s physical effort and active participation in the rehabilitation exercises. Since physiological responses can vary with environmental factors and individuals [41, 42], variation (increases or decreases) of these signals during exercise is considered in the assessment of patient performance and tiredness. Error is a driving signal for human motor relearning in rehabilitation exercises [36]. Therefore, to improve motor relearning, the trajectory tracking error signal, and physiological responses are combined in the implemented system to estimate the patient’s participation during the therapy exercises. In the system, variation of the trajectory tracking error signal is used only for tiredness evaluation. The FIS has three outputs: *performance*, *tiredness*, and *slacking*, as shown in Fig. 3. In the figure, Δ values represent the changes in trajectory tracking error, HR, and GSR signals.

All input and output variables of the FIS are characterized by fuzzy membership (MS) functions and ranged from 0 to 1. The membership functions of the FIS have been defined and characterized based on experimental determination in alignment with existing literature [42, 43]. Inputs can take three values from “low” to “high.” Outputs have five different values as “low,” “midlow,” “medium,” “midhigh,” and “high.” MS functions were regulated experimentally and defined according to the studies in the literature [42, 43]. For establishing the changes in HR, GSR, and the tracking error, these signals are averaged every 5 s of simulation time and compared to their last mean values. If these signals increase or decrease, they take the “high” or “low” value, respectively. The medium value is used for unchanged signals.

**Fig. 4:**
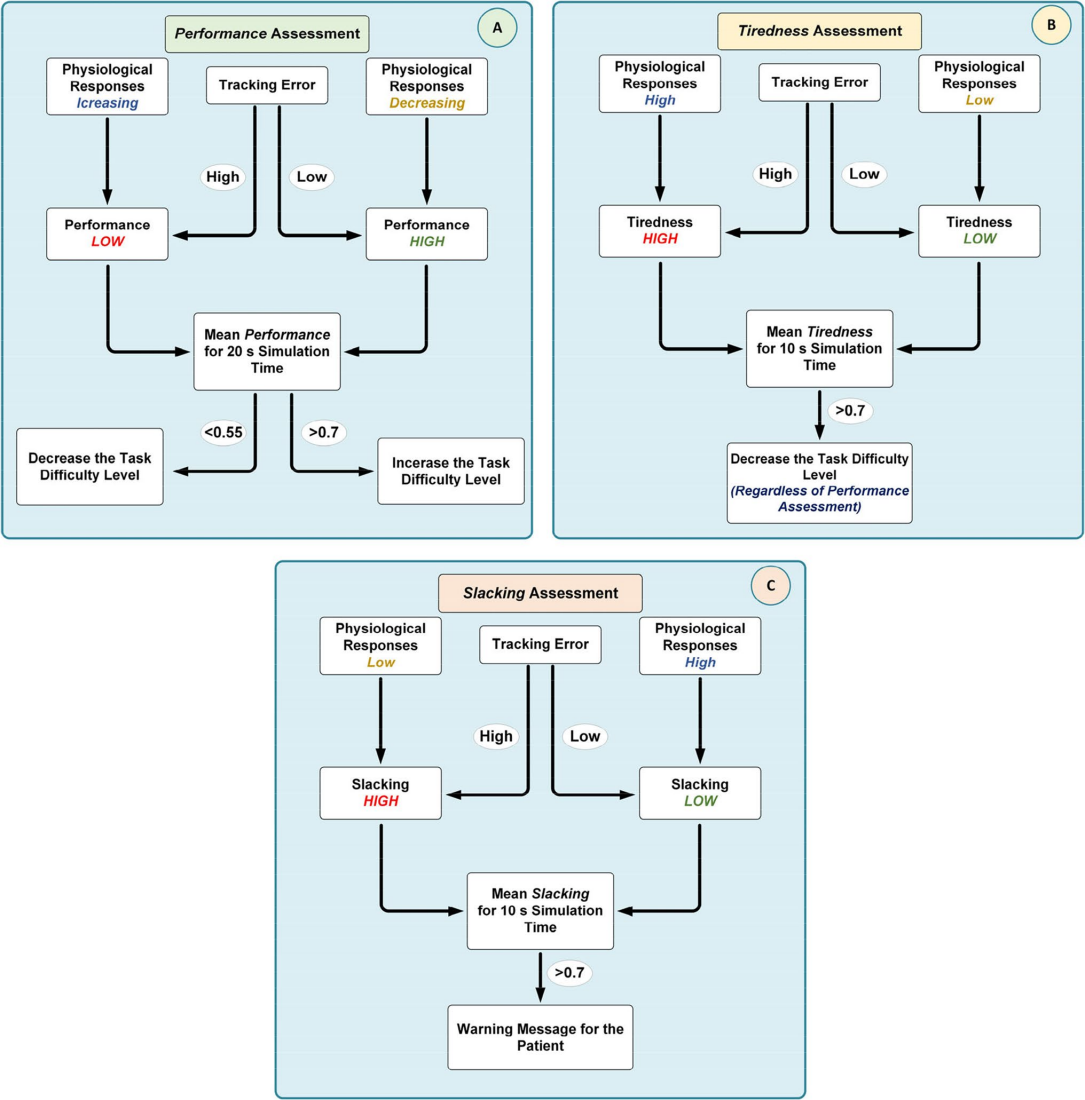
PRES algorithms for **A** performance, **B** tiredness, and **C** slacking assessment and therapy task management process

In the PRES, patients’ therapy performance is evaluated using trajectory-tracking error signals and changes in the physiological responses. Tiredness and slacking are evaluated based on the levels of physiological responses and the error signal. Based on the performance and tiredness outputs of the PRES, the therapy task management module adjusts the therapy tasks and difficulty levels to ensure that the exercise is sufficiently challenging for the patient. The overall structure of the algorithms implemented in PRES and task management module is illustrated in Fig. 4.

For performing the therapy exercises and interacting with the patients, a MATLAB® Graphical User Interface (GUI) is implemented as the rehabilitation interface. To increase motivation, patients are given feedback about their performance, tiredness, and slacking assessment on the rehabilitation interface. The patient’s upper arm and forearm link lengths utilized in upper limb kinematic module calculations can be entered patient-specifically via the interface. Also, the desired trajectory paths are adapted based on the patient’s upper arm and forearm lengths. The details of the implemented system can be found in [37].

### Details of the clinical trials

To demonstrate the effectiveness of the performance evaluation method, the designed system has been tested in clinical trials with patients who have frozen shoulder syndrome. Clinical trials were performed at the private Fizica Medical Center in Adana/Turkey, which has an innovative and multidisciplinary approach to physical medicine and rehabilitation. At this center, there are five specialized doctors and 14 physiotherapists. The facility consists of doctor examination rooms, a special-purpose exercise hall, an exercise hall for neurological and orthopedic rehabilitation, and a fully equipped hand rehabilitation unit under the standards specified by the regulations. Prior to the clinical study, approval was obtained from the clinical trials ethics committee of Çukurova University.

#### Profile of recruited patients

The participants in the clinical study performed were patients diagnosed with frozen shoulder syndrome by a specialist physician. A total of ten patients (two male and eight female) were included in the clinical trials. The recruited patients were aged between 42 and 65; the mean age is 54.7 years, and the standard deviation is 7.9 years. Baseline characteristics of patients are given in Table 1.

**Table 1:** Baseline characteristics of the patients

	Frequency (*n*)	Percentage (%)
Gender		
Female	8	80.0
Male	2	20.0
	Mean ± SD	Med (min–max)
Age (year)	54.7 ± 7.9	53.5 (42–65)
Length (cm)	165.2 ± 8.1	161.5 (159–183)
Weight (kg)	78.2 ± 10.7	76.5 (63–96)
Body mass index (BMI)	28.6 ± 2.2	28.1 (24.6–31.3)

The clinical study excluded patients with the following medical conditions to avoid impacting physiological signal measurements (heart rate and skin conductance) and to accurately assess the system's performance.

- Patients with upper extremity circulation problems
- Patients with pacemakers
- Pregnant patients
- Patients with a history of upper extremity orthopedic surgery in the last six months
- Patients with a chronic disease that may affect balance and coordination

#### Frozen shoulder syndrome: diagnosis and treatment

FSS is a condition of uncertain etiology that causes progressive loss of both active and passive shoulder motion [44]. This syndrome is characterized by pain and limitation in joint range of motion, and the complaints usually regress spontaneously within 1–3 years. However, this period may be prolonged up to 6 years for some patients [45], which diminishes patient comfort and comes with a high socioeconomic cost. Frozen shoulder patients can be divided into two subgroups those with primary frozen shoulder and secondary frozen shoulder [46]. Primary frozen shoulder is a condition seen with a frequency of approximately 2–5% and manifestation with progressive limitation of active and passive shoulder motion. Although it is common in females, it is more frequent between the ages of 30–70 and on the non-dominant side [47]. Besides, its etiology is not known precisely [48]. Secondary frozen shoulder occurs due to intrinsic, extrinsic, and systemic effects, and its possible causes can be trauma and post-surgical process [44, 46]. In this clinical study, all the recruited participants were patients with primary frozen shoulder syndrome.

The diagnosis of frozen shoulder is based mainly on the patient's medical history and clinical examination [46]. Clinically, pain and limitation of movement are the main complaints. Patients commonly report intense pain localized around the deltoid insertion that radiates downward. This pain, associated with FSS, intensifies during the night and prevents the patient's ability to perform ADL.

FSS treatment aims to reduce pain, restore shoulder movement range of motion (RoM), and maintain that motion capability [49]. Although different methods have been applied for therapy, treatments can be divided into two main headings non-surgical and surgical [44]. Non-surgical treatment comprises physical therapy and RoM exercises, drug use, and steroid injections [50]. Surgical treatments involve manipulation under anesthesia, shoulder arthroscopy, and botulinum toxin application.

### Therapy procedure of patients and implementation of participation assessment system

In the clinical trials, the patients followed the treatment protocols recommended by the physician. This therapy protocol includes methods such as joint mobilization, transcutaneous electrical nerve stimulation (TENS) applications, warm modalities, and manipulation that are used in the treatment of primary FSS. The therapy process of the patients contains a total of 20 sessions, 4 weeks and 5 days a week.

During their 4-week treatment period, the participants were asked to perform the RoM exercises using the developed system once a week. RoM exercises were preferred, under the supervision of a specialist physician, because they are used in the treatment of FSS and support the therapy process of the patients [50].

**Fig. 5:**
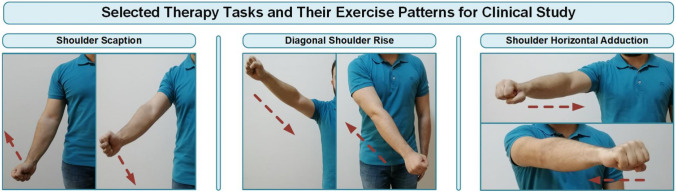
Implemented therapy tasks and their exercise patterns in the clinical study

The selected tasks and their exercise patterns are illustrated in Fig. 5. Each of the selected therapy tasks for clinical study has two different speed levels for the trajectory target. Thereby, a total of six difficulty levels have been defined for three tasks, with two levels for each task.

Before the clinical trials, patients were briefed about the system’s structure, the purpose of the study, and the therapy tasks. At the beginning of the first experiments, the patient was given a few minutes of adaptation time without performing any exercises. After the adaptation period, the patient was given a 5-min break.

The patients performed the exercises with the developed system before the therapy procedures prescribed by the doctor so that the applied treatment methods would not affect the performance evaluation of the system. The applied scales and performed measurements were carried out after the patients completed the exercises with the developed system.

**Fig. 6:**
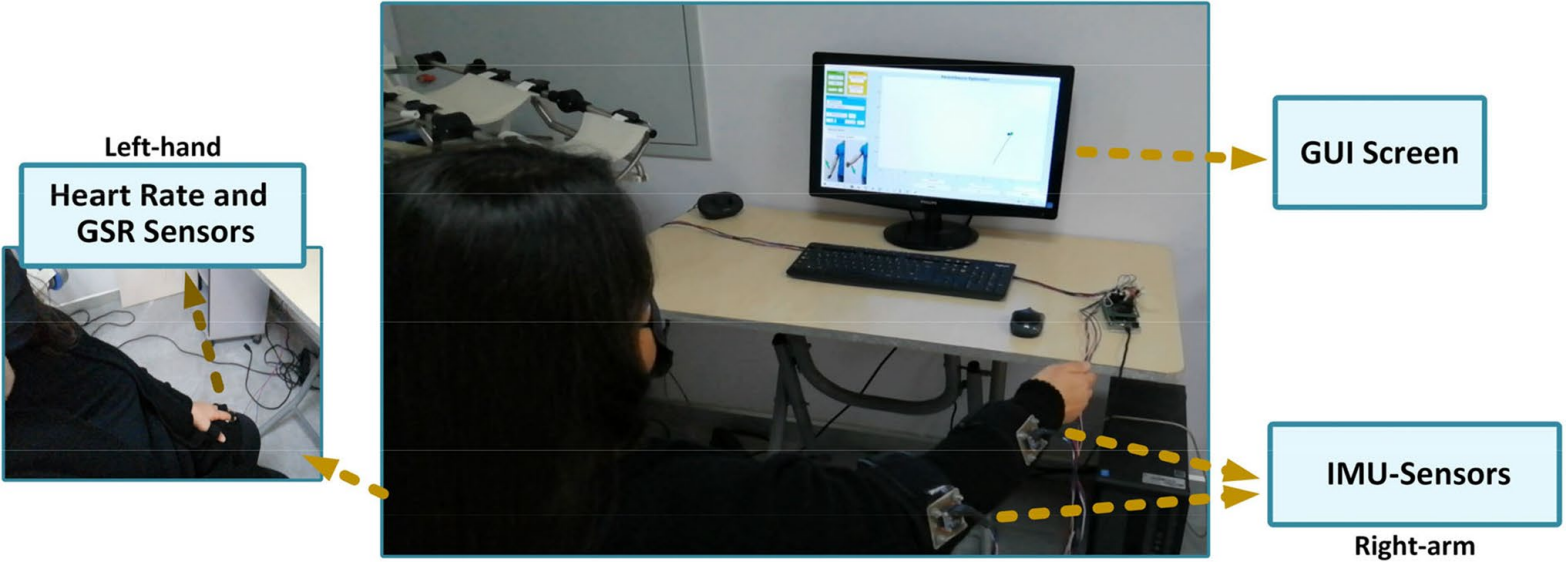
Implementation of the participation assessment system in the clinical environment

The patients performed the experiments by sitting on a chair in front of the GUI screen. The IMU sensor was placed on the participants’ right arm. The HR and GSR sensors were attached to the patient’s left hand. The patients were asked not to move their left hands during the exercises so as not to affect the measurements. The clinical trial implementation of the designed system is shown in Fig. 6. Patients were asked to follow the trajectory target displayed on the GUI screen during the exercises.

### Clinical measurements and implemented scales

Although the implemented system in this study can be used for performing rehabilitation exercises or motion analysis, the system’s primary focus is evaluating patient participation and performance during therapy exercises. Therefore, the clinical study was planned based on comparing the participation and performance assessment data of the designed system with the patient’s progress during the treatment process. In this context, various scales and clinical measurements were carried out to evaluate patients’ improvements during the therapy period and their status during the use of the system.

#### Personal information form

Participants’ demographic information, such as age, height, body weight, and dominant extremity, was collected using the personal information form.

#### Patient self-assessment scale

The self-assessment scale was applied to make patients evaluate their exercise performance. The patients were asked to rate their performance between 0 and 10 after the experiments every week, and the scores given by the patients were recorded.

#### Joint range of motion (RoM) measurement

RoM measurements assist physicians in making diagnoses, determining functional limitations, monitoring therapy improvements, or demonstrating treatment outcomes. Therefore, in clinical and scientific research, RoM measurements are performed using many methods, from simple tapes to electrical goniometers and kinematic analysis systems that evaluate patient kinematic data [51]. In this study, the patients’ shoulder joint RoM angles were measured by a clinician after weekly experiments and recorded. A digital inclinometer/goniometer was used for RoM measurements.

#### Visual analog scale for physical fatigue and pain

Visual analog scale (VAS) is used to convert some values that cannot be measured numerically into quantitative. This scale consists of a 10-cm horizontally or vertically positioned line. At both ends of the line, there are two contrary statements about the situation to be evaluated. In the scales used in this study, these conditions are “extreme tiredness,” “not feeling tired” for the fatigue scale, “having no pain,” and “unbearable pain” for the pain assessment. During clinical trials, participants were asked to mark the point on the VAS line that best reflected their fatigue and physical pain level. The point marked by the patient is measured from the lower end of the line with the help of a ruler in cm to determine the patient’s fatigue and pain score. VAS is a valid and reliable method for fatigue and pain assessment [52, 53].

### Statistical analysis

Statistical analysis was performed to evaluate the findings obtained from clinical trials using the SPSS (Statistical Package for the Social Sciences) 25.0 software. In the analysis, the averages of the patients’ data according to the weeks were used. Categorical measurements were summarized as numbers and percentages, and continuous measurements as mean and standard deviation (median and minimum–maximum where appropriate). Shapiro–Wilk test was used to determine whether the parameters in the study showed a normal distribution. The repeated measures ANOVA test was used to examine the difference between the data according to weeks. Wilcoxon rank test was used to analyze the difference between weekly variations. For examining the relationship between continuous measurement parameters, the Spearman correlation test was implemented. The statistical significance level was taken as *p* < 0.05 in all tests.

## Results

**Fig. 7:**
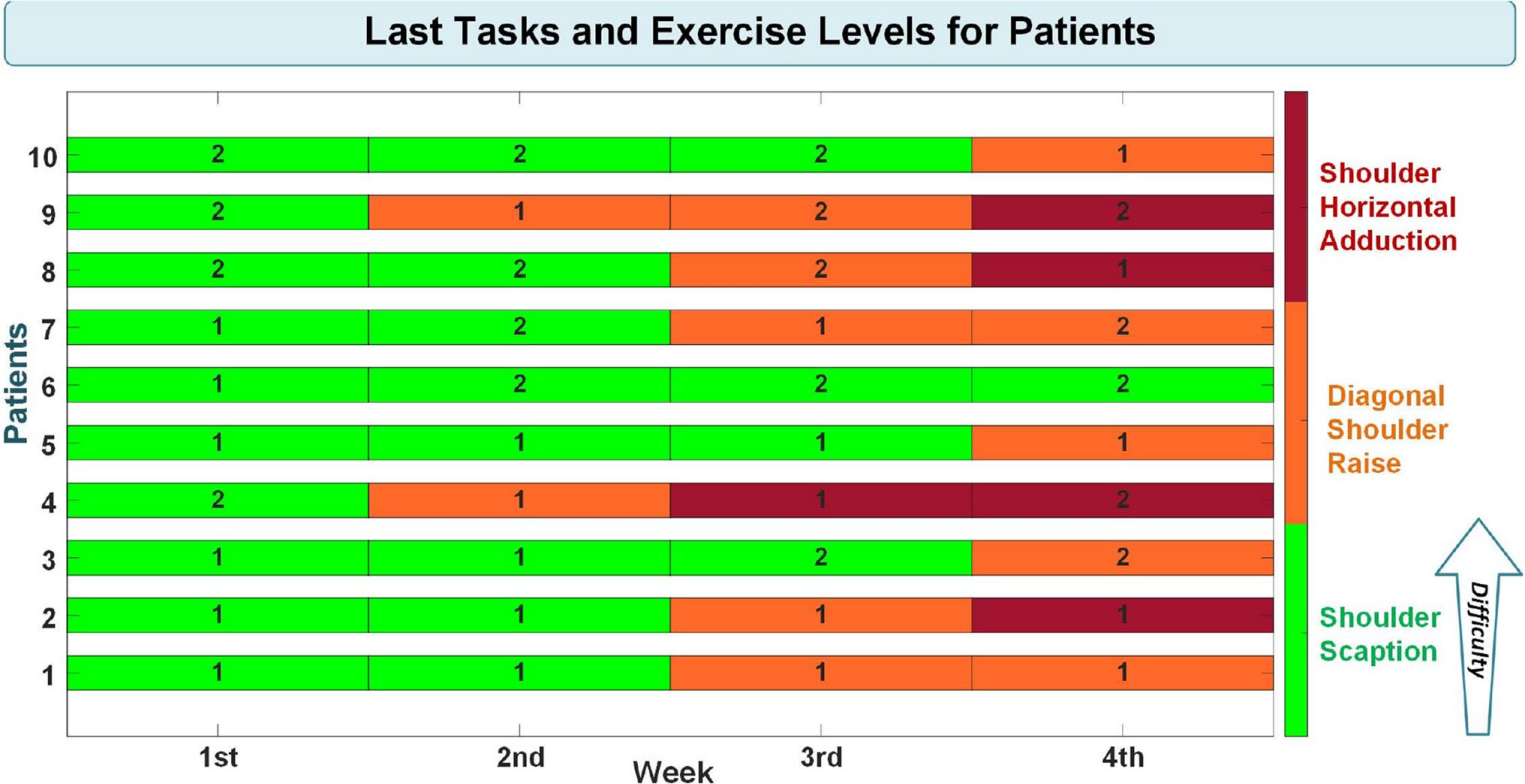
Patients’ last tasks and exercise levels during the four-week experiments

The participants exercised for between 5 and 7 min with the developed system during the clinical study, depending on their performance. All experiments were carried out under the supervision of a physiotherapist. The initial exercise level is always shoulder scaption — first speed level of the therapy target. The task levels at which the patients completed the exercises during the 4-week experiments are given in Fig. 7.

The boxes in the figure show at which task level the patients completed the experiments that week. The numbers on the boxes represent the exercise level of the therapy task. During the 4-week experiments, two patients reached the last task and exercise level. Two participants reached the final task level but not the final exercise level. Five patients completed the experiments at the second task level, and one patient finished the experiments at the first task level.

Table 2 shows the weekly changes in the shoulder joint RoM angles of the patients. When the findings were examined, it was determined that the weekly changes in shoulder flexion (*p* < 0.001), shoulder extension (*p* < 0.001), shoulder abduction (*p* < 0.001), shoulder adduction (*p* < 0.001), shoulder internal rotation (*p* < 0.001), and shoulder external rotation (*p* < 0.001) were statistically significant (*p* < 0.01).

**Table 2:** Examining the weekly changes in the shoulder joint angles

	1. Week	2. Week	3. Week	4. Week	*p*
	Mean ± SD	Mean ± SD	Mean ± SD	Mean ± SD	
Shoulder flexion	120.8 ± 19.5°	130.3 ± 18.8°	139.1 ± 5.9°	147.1 ± 13.6°	< 0.001**
Shoulder extension	22.4 ± 4.7°	29.8 ± 6.8°	35.7 ± 5.9°	40.6 ± 5.9°	< 0.001**
Shoulder abduction	103.0 ± 18.1°	119.0 ± 21.2°	131.2 ± 19.1°	139.9 ± 14.5°	< 0.001**
Shoulder adduction	11.0 ± 3.8°	18.0 ± 3.9°	23.5 ± 3.7°	27.2 ± 2.7°	< 0.001**
Shoulder internal rotation	51.3 ± 15.2°	57.8 ± 16.9°	68.3 ± 14.4°	74.0 ± 14.2°	< 0.001**
Shoulder external rotation	40.5 ± 18.3°	47.0 ± 19.1°	58.4 ± 17.4°	63.8 ± 15.9°	< 0.001**

**p < 0.01, repeated measures ANOVA test

Also, the differences between the weekly changes in shoulder joint angles were examined in Table 3 and it was seen that there was a significant difference between the weekly variations.

**Table 3:** Differences between weekly changes in shoulder joint angles

	p1	p2	p3	p4	p5	p6
Shoulder flexion	0.005**	0.005**	0.005**	0.005**	0.005**	0.005**
Shoulder extension	0.005**	0.004**	0.005**	0.005**	0.005**	0.007**
Shoulder abduction	0.008**	0.005**	0.005**	0.005**	0.005**	0.005**
Shoulder adduction	0.007**	0.005**	0.005**	0.005**	0.005**	0.007**
Shoulder internal rotation	0.007**	0.005**	0.005**	0.011*	0.008**	0.007**
Shoulder external rotation	0.007**	0.005**	0.005**	0.005**	0.005**	0.007**

*p < 0.05, **p < 0.01; Wilcoxon rank test, p1: 1. week—2. week, p2: 1. week -3. week, p3: 1. week—4. week, p4: 2. week—3. week, p5: 2. week—4. week, p6: 3. week—4. week

VAS pain (VASP) scores of the patients were statistically analyzed according to the weeks, and it was observed that the mean VASP scores of the patients decreased significantly (Table 4). In addition, the weekly variations in the patients’ VASP scores, seen in Table 5, were also statistically significant.

**Table 4:** Examination of weekly changes in patients’ VAS pain scores

VAS	1. Week	2. Week	3. Week	4. Week	*p*
	Mean ± SD	Mean ± SD	Mean ± SD	Mean ± SD	
Pain (VASP)	5.87 ± 0.9	4.84 ± 0.9	3.54 ± 1.2	2.19 ± 0.6	< 0.001**

**p < 0.01; repeated measures ANOVA test

**Table 5:** Differences between patients’ weekly changes in VAS pain scores

VAS	p1	p2	p3	p4	p5	p6
Pain (VASP)	0.005**	0.005**	0.005**	0.007**	0.005**	0.005**

**p < 0.01; Wilcoxon rank test, p1: 1. week—2. week, p2: 1. week -3. week, p3: 1. week—4. week, p4: 2. week—3. week, p5: 2. week—4. week, p6: 3. week—4. week

**Fig. 8:**
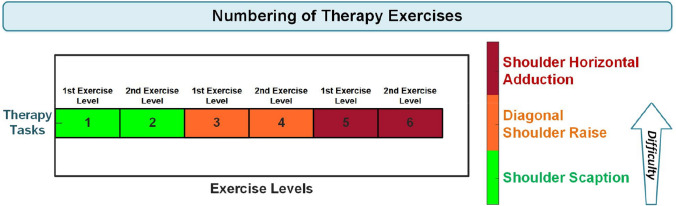
Numbering of therapy tasks and exercise levels

Data obtained from the implemented performance assessment system were analyzed statistically additively to the scales applied to the patients. As the data acquired from the system, the average weekly performance of the patients evaluated by the developed system and the task levels at which the patients completed the exercises according to weeks, by numbering the therapy tasks specified in Fig. 5, was used. This numbering was performed by evaluating the first therapy task and exercise level (shoulder scaption — first-speed level of the therapy target) as one and the last task and exercise level (shoulder horizontal adduction — second-speed level of the therapy target) as six. The numbering process is depicted in Fig. 8. The numbers displayed on the boxes indicate the exercise levels.

The results of the analysis show that the weekly average performance of the patients increased significantly (Table 6). Moreover, weekly variations of the average system performance evaluation values of the patients were also found to be significant (Table 7).

**Table 6:** Examining the weekly changes in the mean performances of the patients evaluated by the developed system

	1. Week	2. Week	3. Week	4. Week	*p*
	Mean ± SD	Mean ± SD	Mean ± SD	Mean ± SD	
System performance evaluation (SPE)	0.58 ± 0.03	0.60 ± 0.03	0.63 ± 0.03	0.66 ± 0.03	< 0.001**

**p < 0.01; repeated measures ANOVA test

**Table 7:** Differences between weekly changes in the mean performance of patients assessed by the system

	p1	p2	p3	p4	p5	p6
System performance evaluation (SPE)	0.005**	0.005**	0.005**	0.005**	0.005**	0.005**

**p < 0.01; Wilcoxon rank test, p1: 1. week—2. week, p2: 1. week -3. week, p3: 1. week—4. week, p4: 2. week—3. week, p5: 2. week—4. week, p6: 3. week—4. week

In addition to the increase in the system-evaluated performance of the patients, there was a significant increase in the therapy levels at which they completed the exercises during the clinical trials (Table 8). Also, as seen in Table 9, the differences between weekly changes in the exercise levels of the patients are significant.

**Table 8:** Examining the weekly changes in patient exercise levels

	1. Week	2. Week	3. Week	4. Week	*p*
	Mean ± SD	Mean ± SD	Mean ± SD	Mean ± SD	
Patients exercise levels (PEL)	1.40 ± 0.5	1.80 ± 0.8	2.90 ± 1.2	4.10 ± 1.4	< 0.001**

**p < 0.01; repeated measures ANOVA test

**Table 9:** Differences between weekly changes in patient exercise levels

	p1	p2	p3	p4	p5	p6
Patients exercise levels (PEL)	0.046*	0.010*	0.005**	0.015*	0.007**	0.010*

*p < 0.05, **p < 0.01; Wilcoxon rank test, p1: 1. week—2. week, p2: 1. week -3. week, p3: 1. week—4. week, p4: 2. week—3. week, p5: 2. week—4. week, p6: 3. week—4. week

No slacking was detected for any patient during the clinical trials. Also, most of the patients had low scores on VAS fatigue (VASF) during the experiments, and there was generally no significant change in their scores throughout the weeks. The weekly changes in the VASF scores of the patients and differences between weekly variations are examined in Table 10 and 11, respectively.

**Table 10:** Examination of weekly changes in patients’ VAS fatigue scores

VAS	1. Week	2. Week	3. Week	4. Week	*p*
	Mean ± SD	Mean ± SD	Mean ± SD	Mean ± SD	
Fatigue (VASF)	1.10 ± 0.2	1.58 ± 1.7	0.96 ± 0.3	0.91 ± 0.2	0.026*

*p < 0.05; repeated measures ANOVA test

**Table 11:** Differences between patients’ weekly changes in VAS fatigue scores

VAS	p1	p2	p3	p4	p5	p6
Fatigue (VASF)	0.325	0.137	0.032*	0.091	0.049*	0.343

*p < 0.05; Wilcoxon rank test, p1: 1. week—2. week, p2: 1. week -3. week, p3: 1. week—4. week, p4: 2. week—3. week, p5: 2. week—4. week, p6: 3. week—4. week

**Fig. 9:**
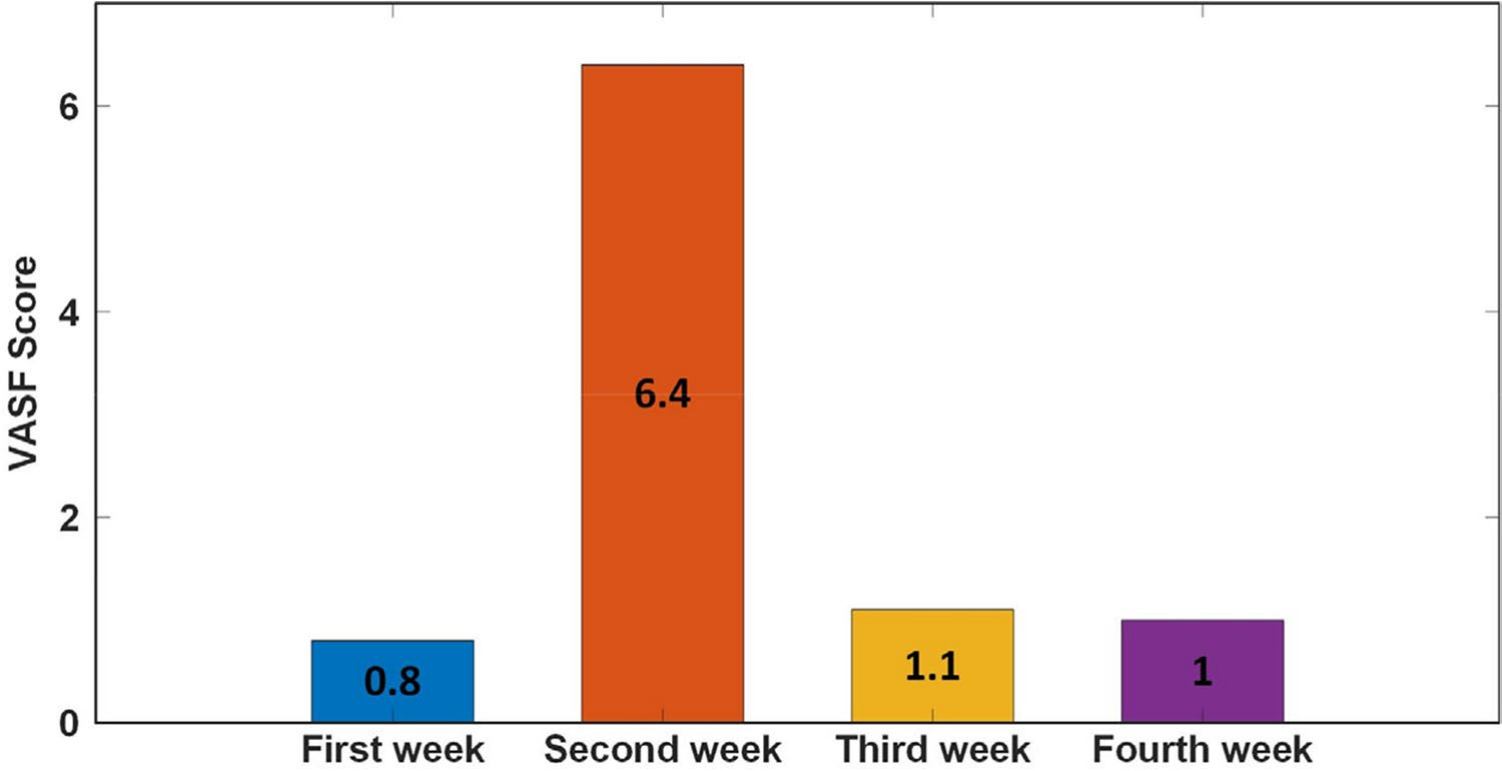
Weekly VAS fatigue scores of the second patient

When the weekly VASF scores of the patients given in Table 10 are examined, an increase is observed in the average of the second week’s VASF score. The main reason for this increase is that the VASF score of the second patient this week is higher than the values in the other weeks. The second patient’s weekly VASF scores are given in Fig. 9.

**Fig. 10:**
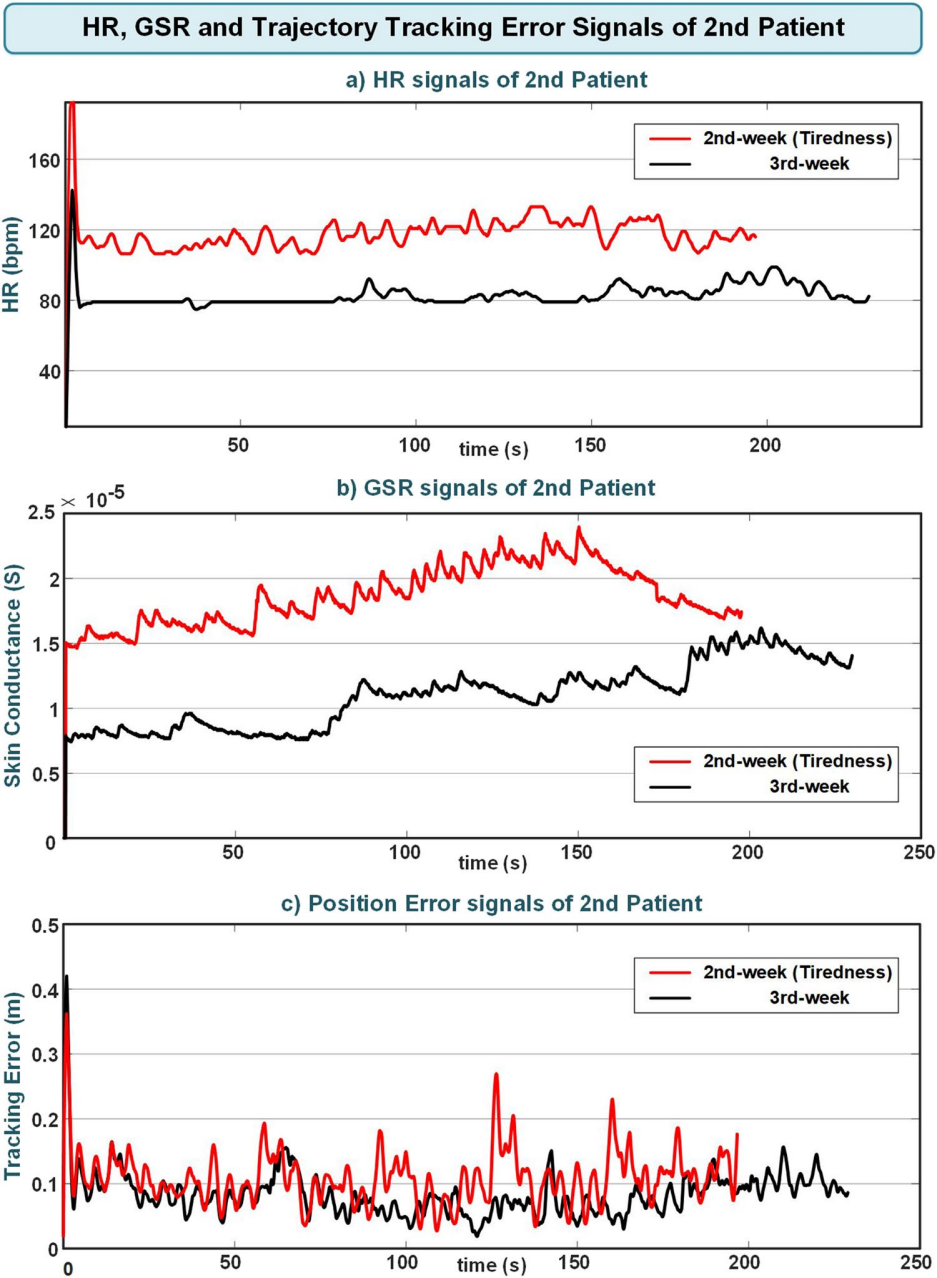
Second patient’s **a** HR, **b **GSR, and **c** trajectory tracking error signals during the second-week and third-week experiments

In addition, tiredness was detected by the developed participation evaluation system during this patient’s second week of experiments. HR, GSR, and trajectory tracking error signals of this patient during the exercises in the second week in which tiredness was detected and the signals in the third week when there was no tiredness detection are given together in Fig. 10 for comparison.

**Fig. 11:**
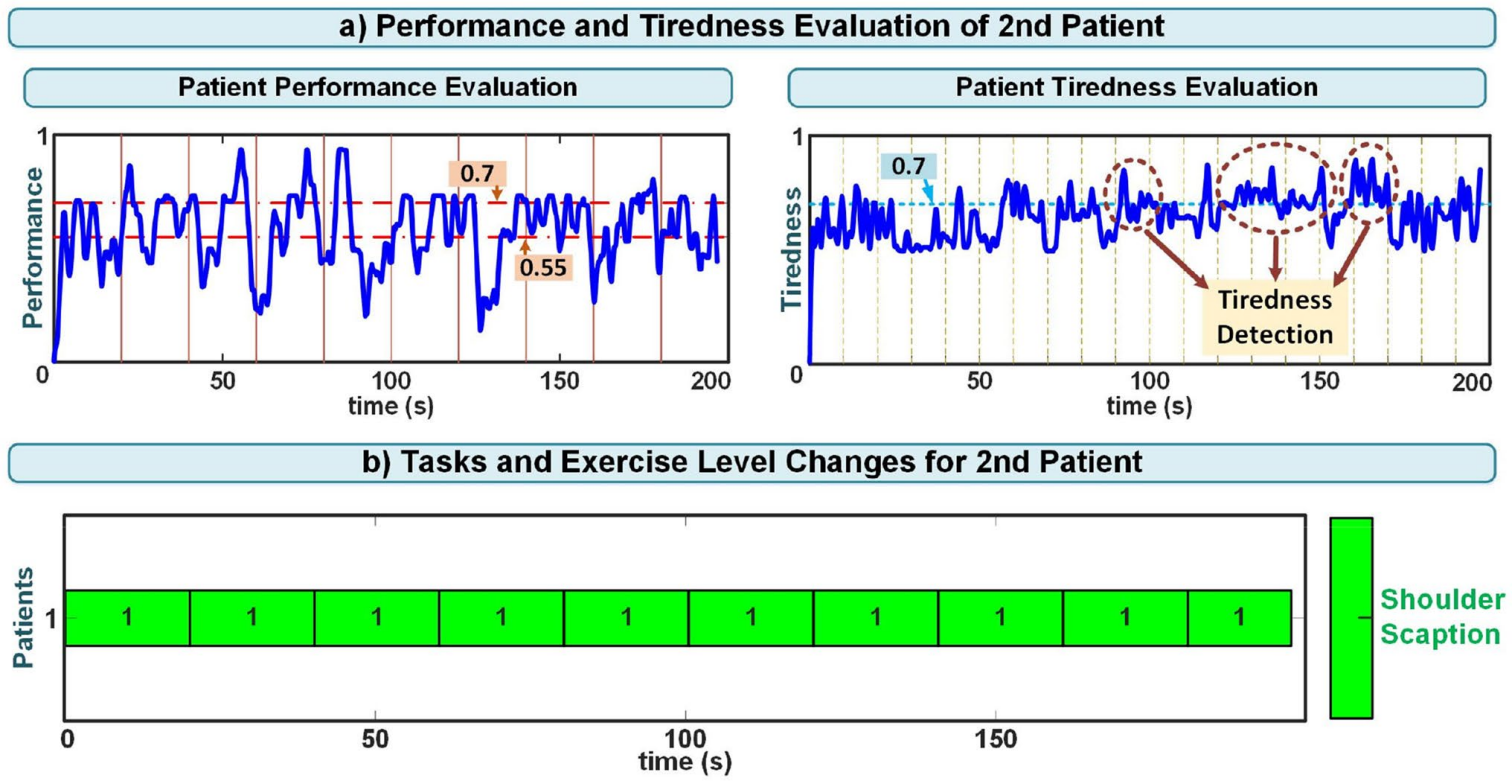
Second patient’s performance and tiredness evaluation and exercise level changes during the second-week experiment

When examining the physiological signals of this patient, it was observed that the physiological signals during the second week, when fatigue was detected, were higher compared to the signals in the third week. Additionally, the trajectory tracking error signal was greater in the second week than in the third week. In addition to all of these findings, the patient reported feeling tired following the experiments conducted this week. The performance, tiredness assessments, and variations in task difficulty levels of the patient during the second-week experiment are given in Fig. 11. The vertical lines seen in section (a) of the figure indicate the intervals at which the patient’s performance and fatigue were evaluated. The values of 0.7 and 0.55, presented in the patient performance evaluation section of the figure, respectively, denote the upper and lower limits for increasing or decreasing exercise levels based on performance assessment. The values of 0.7 in the patient tiredness evaluation section represent the upper limit for reducing the exercise level depending on the tiredness assessment. These upper and lower limits in performance and fatigue assessments have been experimentally determined.

Based on the patient’s high physiological responses and the trajectory tracking error signal, tiredness was detected by the implemented system, and the patient’s exercise level remained constant at the initial level during the experiments of the second week. This tiredness detection is consistent with the patient’s VASF score and reveals that the system can successfully evaluate the patient’s fatigue.

Patients’ self-assessment (PSA) scores during the clinical trials are given in Table 12. In the analysis, the weekly change in patient self-assessment scores was found to be statistically significant (*p* < 0.001). Also, the differences between the weekly changes in the patient self-assessment scores were examined in Table 13.

**Table 12:** Examination of weekly changes in patient self-assessment scores

	1. Week	2. Week	3. Week	4. Week	*p*
	Mean ± SD	Mean ± SD	Mean ± SD	Mean ± SD	
Patient self-assessment (PSA)	7.10 ± 1.3	7.35 ± 1.1	8.15 ± 0.9	9.4 ± 0.7	< 0.001**

**p < 0.01; repeated measures ANOVA test

**Table 13:** Differences between weekly changes in patient self-assessment scores

	p1	p2	p3	p4	p5	p6
Patient self-assessment (PSA)	0.595	0.010*	0.004**	0.042*	0.004**	0.006**

*p < 0.05, **p < 0.01; Wilcoxon rank test, p1: 1. week—2. week, p2: 1. week -3. week, p3: 1. week—4. week, p4: 2. week—3. week, p5: 2. week—4. week, p6: 3. week—4. week

## Discussion

In this study, the effectiveness of the participation assessment system, which evaluates patients’ therapy performance, tiredness, and slacking, was tested with patients suffering from frozen shoulder syndrome, a condition that causes pain in patients and makes ADL difficult by restricting shoulder joint movements [46]. Although this syndrome can be seen in all age groups, it is more common between the ages of 40–60 and in females [44, 47]. These findings are consistent with the mean age of the patients participating in the study and the proportion of females among the patients.

During the clinical trials, slacking was not detected in any of the patients. This finding can be interpreted as patients who come to a private clinic by paying a fee to be treated actively participate in therapy exercises. Also, the absence of any assistive device usage during the experiments might have prevented the emergence of slacking behavior. In addition, in the correlation tests, no significant correlation was found between the changes in the joint angles of the patients and the BMI and age data.

The majority of patients’ VASF scores during the experiments were low and did not show an overall significant change over the weeks (Table 10 and 11). However, as seen in Fig. 9, the second patient had a high VASF score during the second-week experiments. Additionally, the developed system detected fatigue based on increased physiological responses and elevated trajectory tracking error signals during the patient’s exercises this week (Fig. 11). The system’s tiredness detection aligns with the patient’s VASF score, indicating the system’s capability to effectively assess the patient’s fatigue.

When the shoulder joint angles and VASP scores of the patients were examined with the repeated measures test, it was observed that the shoulder joint angles increased each week significantly during the treatment, while the VASP scores decreased significantly (Table 2 and 4).

As shown in Table 6 and 8, the weekly performances of the patients evaluated by the system and the levels at which they completed the exercises significantly increase each week. In addition, as seen in Table 12, PSA scores commonly increase significantly. Evaluating PSA scores alongside patient performance assessments and exercise levels, it can be concluded that patients can correctly evaluate their progress but give high scores when rating their therapy performance.

In the correlation tests, no significant relationship was found between the changes in the shoulder joint angles and VASP scores of the patients, and the changes in the patient’s performance evaluations and exercise levels. It is considered that these results are due to the differences in the weekly rate of change of the findings. In treating frozen shoulder syndrome, the main criteria for recovery are reduced pain and increased joint angles [45]. The primary joint limitations observed in frozen shoulder patients include shoulder flexion of less than 120° and more than a 50% decrease in external rotation [46]. When examining the findings of the patients involved in the study before the treatment, it is seen that shoulder flexion is at this limit, while the external rotation has decreased by greater than 50% [46, 54]. At the end of the therapy process, it is observed that the patients’ RoM values for both movements have improved, exceeding the typical values seen in frozen shoulder patients (Table 2). Additionally, in terms of pain, studies in the literature show that a three-point or 33% decrease in the VAS pain score is significant for patients [55, 56]. The changes in patients’ VASP scores during the treatment process align with these findings and are meaningful (Table 4). When clinically examining patient performance and exercise level data with these findings, it is observed that the results are consistent with weekly changes in patient performance assessments and exercise levels. While joint angles increased and VASP scores decreased as an indicator of patient improvement and progress, patients’ performance evaluation values and exercise levels increased in parallel. These findings reveal that the developed system can successfully evaluate patient performance clinically.

## Limitations

Despite the satisfying results obtained from clinical trials, the number of patients limits the generalizability of the results. The clinical study was performed during the COVID-19 pandemic, and it is considered that the reluctance of patients to go to medical institutions during the pandemic is effective in the low number of participants [57]. Furthermore, fatigue that emerges during physical exertion is impacted by various elements such as age, gender, fitness history, and the type of workout [58]. Therefore, clinical studies with a larger patient population may provide a more comprehensive evaluation of the developed system’s effectiveness. The fuzzy membership functions and rule set in the proposed method were established through experimental determination. While the experimental outcomes demonstrate the successful functioning of the designed FIS, this approach might impact the system’s performance estimation.

## Conclusions

In this paper, a patient participation assessment system, which can evaluate patient’s therapy participation independent of any device design and therapy exercise has been clinically tested. The implemented system assesses the patient’s performance, tiredness, and slacking during the rehabilitation exercises depending on physiological responses and trajectory tracking error signals. Also, the system adjusts therapy tasks and task difficulty based on the participation of the patient. The clinical study involved ten patients diagnosed with FSS. Throughout the 4-week treatment period, the patients engaged in three different RoM exercises while using the participation assessment system, additively to their treatment protocols. These exercises were performed once a week, and due to the system being independent of hardware, no additional therapy devices were used. During the clinical trials, multiple measurements and scales were utilized to assess the progress of patients throughout the treatment process. The clinical findings and data obtained from the applied participation assessment system were statistically analyzed and compared to demonstrate the effectiveness of the system in evaluating patients’ performance, tiredness, and slacking.

The findings from the clinical study revealed that patients’ progress and recovery during the therapy period coincided with the weekly changes in the system’s performance evaluation and the patients’ exercise levels. In addition, the designed system successfully detected patient tiredness during the clinical trials. These results show that the applied participation assessment system successfully evaluated patients’ performance and tiredness during rehabilitation exercises in a clinical setting, independent of any device design and therapy task.
